# Supplementary material for: DeLTA 2.0: A deep learning pipeline for quantifying single-cell spatial and temporal dynamics
Source: PLoS Comput Biol. 2022 Jan 18;18(1):e1009797. doi: 10.1371/journal.pcbi.1009797 (PMC8797229; doi:10.1371/journal.pcbi.1009797)
Supplement: S1 Table — Package name and respective number of the version that was used for analysis presented in this manuscript, as well as for other working installations. (DOCX) [file pcbi.1009797.s012.docx]

Linux – conda yml install, TF 2.4 (reference installation for this manuscript)

| **Package Name** | **Version** |
| --- | --- |
| python | 3.7.10 |
| spyder | 5.1.5 |
| scikit-image | 0.18.3 |
| tifffile | 2021.8.30 |
| opencv | 4.5.2 |
| tensorflow-gpu | 2.4.1 |
| ffmpeg-python | 0.2.0 |
| git | 2.33.0 |
| pip | 21.2.4 |
| python-bioformats | 4.0.5 |

Linux – conda yml install, TF 2.0

| **Package Name** | **Version** |
| --- | --- |
| python | 3.7.0 |
| spyder | 5.1.5 |
| scikit-image | 0.18.3 |
| tifffile | 2021.7.2 |
| opencv | 4.5.3 |
| tensorflow-gpu | 2.0.0 |
| ffmpeg-python | 0.2.0 |
| git | 2.20.1 |
| pip | 21.3.1 |
| python-bioformats | 4.0.5 |

Windows 10 pip install – TF 2.7

| **Package Name** | **Version** |
| --- | --- |
| python | 3.8.12 |
| scikit-image | 0.18.3 |
| tifffile | 2021.11.2 |
| opencv | 4.5.4.58 |
| tensorflow | 2.7.0 |
| ffmpeg-python | 0.2.0 |
| pip | 21.0.1 |
| python-bioformats | 4.0.5 |

Windows 10 conda yml install -TF 2.5

| **Package Name** | **Version** |
| --- | --- |
| Python | 3.9.7 |
| spyder | 5.1.5 |
| scikit-image | 0.18.3 |
| Tifffile | 2021.10.12 |
| Opencv | 4.5.1 |
| tensorflow-gpu | 2.5.0 |
| ffmpeg-python | 0.2.0 |
| Git | 2.33.1 |
| Pip | 21.3.1 |
| python-bioformats | 4.0.5 |

Windows 10 conda yml install – TF 2.3

| **Package Name** | **Version** |
| --- | --- |
| python | 3.8.8 |
| spyder | 5.0.0 |
| scikit-image | 0.18.1 |
| tifffile | 2021.3.17 |
| opencv | 4.5.1 |
| tensorflow-gpu | 2.3.0 |
| ffmpeg-python | 0.2.0 |
| git | 2.30.2 |
| pip | 21.0.1 |
| python-bioformats | 4.0.4 |
