# Supplementary material for: DeLTA 2.0: A deep learning pipeline for quantifying single-cell spatial and temporal dynamics
Source: PLoS Comput Biol. 2022 Jan 18;18(1):e1009797. doi: 10.1371/journal.pcbi.1009797 (PMC8797229; doi:10.1371/journal.pcbi.1009797)
Supplement: Attachment — Submitted filename: Response_to_Reviewers.docx [file pcbi.1009797.s013.docx]

**PCOMPBIOL-D-21-01464**

**Point-by-Point Response to Reviewers**

We thank the reviewers for their comments and suggestions for improving the content and clarity of the manuscript. We have addressed all their concerns in the revised manuscript. In particular, we feel that the suggestions and feedback from the reviewers to improve our versioning, simplify installation, provide small sample datasets, and to simplify data downloads have greatly improved the accessibility of our software. We now provide DeLTA 2.0 as both a pip and conda-forge package that can be installed in one single line, we have added a module to automatically download our latest assets such as trained models or training sets, we provide a Google Colab notebook for potential users to rapidly try DeLTA’s performance on their data, and we provide new, extensive documentation to help users install and run DeLTA 2.0.

A complete list of changes and detailed responses to the comments follows.

**Reviewer #1:**

O’Connor et al describe DeLTA 2.0, a cell-segmentation, tracking, and fluorescence data analysis pipeline which improves upon the original version of DeLTA in several significant ways. In the introduction, the authors note that increasing data throughput requires reducing time spent on manually correcting segmentation and tracking. This undersells the potential impact of this work, which does not require particularly high throughput data to save many hours. There are also experiments beyond timelapse fluorescence microscopy of bacteria that the DeLTA 2.0 data pipeline can be modified to cover. And some examples the authors cite include a constitutive fluorescence signal to assist in segmentation, which is a complication that can hopefully be avoided with DeLTA.

We appreciate these points and have added text in the Introduction and Discussion related to this.

🡪 See Introduction and Discussion

In addition to creating an end-to-end pipeline from raw data to fluorescence for cell lineages, DeLTA 2.0 makes improvements to segmentation training that increase training speed and accuracy. The authors show that the network can be reduced in size without much impact on performance, which would increase the number of potential users with mobile GPUs, for example, or allow for larger images to be processed without tiling.

As with DeLTA 1.0, in my hands DeLTA 2.0 must be trained on our particular data sets to achieve usable segmentation; despite having a similar resolution, a reduction in contrast in our images requires retraining the network. This is in contrast with MiSiC (DOI: 10.7554/eLife.65151) that applies a shape index map to all training and prediction inputs to achieve a degree of generalization. In future work, it would be interesting to see whether a DeLTA 2.0 model trained on shape index maps would be sufficient to generate initial training data.

One of our core principles in writing the code has been to avoid introducing pre- or post-processing operations and rules to our analysis pipeline to the greatest extent possible. Our rationale for this is that these additions will reduce the flexibility for applications to different use cases, such as new organisms or fluorescence images. Instead of programmed rules, we want most of the work to be done by the U-Nets themselves as they can be re-trained, and data augmentation operations can help generalize to new data. Further, shape index maps can add parameters that the user will need to optimize or that need to be hard-coded. In the case of MiSiC, the sigma parameter of the skimage.feature.shape_index call is hard-coded with 3 values: 1, 1.5, and 2. While we agree that operations like this may improve performance for specific cases, we have opted here instead for training-based solutions. We have clarified this implementation decision in the text.

🡪 See Introduction and Discussion

For many labs the barrier to using DeLTA will be in generating training data and adjusting training parameters. In the readme file the authors write, “We are currently working on the implementation graphical interfaces to generate training sets and to analyze and correct delta outputs,” which will be important, especially for those without Matlab.

Specific comments:

* Line 141: “DeLTA 2.0 can process datasets of any dimension quickly” is presumably not true at some limit. A brief discussion of system and GPU memory requirements and likely performance on a common consumer-grade processor and GPU would be useful. On a PC with 16 GB of system memory I ran into some issues for a large example file.

We have clarified that DeLTA can process datasets of various dimensions, but that there are practical limits. We give an example of a large time-lapse movie that may cause memory issues on some systems. We also now include results from a computationally expensive time-lapse movie that takes DeLTA over an hour to process as an example.

🡪 See Results and S2 Movie

We also recently identified a bug that caused GPU memory issues on Windows with recent versions of Tensorflow (2.6+), even on relatively small files. We enforce a limit on the number of inputs processed at once as a bug fix.

* Line 193: The addition of noise is also a feature of MiSiC - 10.7554/eLife.65151 - which could be discussed further. With DeLTA 1.0, I found that the inverse operation also worked – applying a low-pass filter to training and test data improved performance, especially for segmenting objects thinner than E. coli cells. My impression was that this occurred because some data augmentation operations effectively do this by interpolating pixels, so most training data was unlike test data.

In our suite of data augmentations, we use a Gaussian blur function (low-pass filter) as well as Gaussian noise. However, we do not add noise to our data as a preprocessing step prior to prediction. MiSiC does so because, using a shape index map with fixed sigma values, they sometimes get a preprocessed map that is too smooth compared to what their U-Net is used to segmenting, especially when images are resized, which leads to over-segmentation. We chose to avoid pre-processing steps for prediction as much as possible. For DeLTA, we recommend that data augmentation operations combined with a diverse training set should be used instead to make analysis generalizable.

🡪 See Introduction and Discussion

* Figure 2 and Figure S1: I do not see any need for Fig. S1; the difference in growth rates is very clear in Fig. 2D as well as Fig. A. Also, the unlabeled cells in the “Susceptible” panel of Fig. 2B appear to be “Resistant” and perhaps it would be more clear to show only this microcolony, highlighting the green “Susceptible” cell and purple “Resistant” cells in all frames.

We have removed Fig. S1.

We have also replaced the images of the “Susceptible” cells with another field of view where resistant cells do not grow into the frame. We agree this was confusing and thank the reviewer for pointing it out.

🡪 See Fig. 2B

General minor comments:

* The authors could briefly compare to DeepCell - 10.1371/journal.pcbi.1005177 - which also describes E. coli segmentation and tracking.

We have added this reference in the manuscript.

🡪 See Introduction

* Installation on Windows is not straightforward, but is described well enough in the readme to manage. Listing specific versions in the .yml file would have been helpful, and perhaps installation can be simplified for users not needing bioformats.

We added a supplementary table (S1 Table) that lists the exact versions used during installation for this manuscript. We also added a page in the documentation that we will keep up to date with recent, successful environments.

🡪 See S1 Table

🡪 See <https://delta.readthedocs.io/en/latest/usage/installs_lists.html>

* The file including models, example data, and training data is very large. Separate files would be useful--especially a small file of example data.

We agree with the reviewer that data management was previously tedious, and note that Reviewer #2 raised a similar issue. We now provide small test movies in the DeLTA git repository in our tests folder. We also wrote a new module, assets.py, that automatically and selectively downloads our latest trained models as well as training and evaluation datasets, and updates configuration files accordingly. This support module is described in the documentation.

🡪 See Gitlab

🡪 See <https://delta.readthedocs.io/en/latest/usage/assets_desc.html>

* More people will try DeLTA if there is a Google Colab notebook or similar that eliminates needing to sort out system configuration; it would be valuable to have one example notebook for training and one example notebook for segmentation, tracking, and displaying some figures of timelapse data similar to those in the paper.

We strongly agree and note that this was also suggested by Reviewer #2. To address this, we wrote a Colab notebook with multiple examples to run our main pipeline on our data as well as on the user’s own movie files. A link to the notebook is now provided in the text and in the documentation:

<https://colab.research.google.com/drive/1UL9oXmcJFRBAm0BMQy_DMKg4VHYGgtxZ>

We also added a training section to the notebook, however on the free tier of Google Colab we ran into resource limitations issues. We provide training scripts in our git repository that can be run directly after installing DeLTA and downloading assets, which hopefully will be an easier process now.

🡪 See <https://delta.readthedocs.io/en/latest/usage/scripts.html>

Finally, we provide a few examples in the documentation on how to use the output files for further analysis and making figures.

🡪 See <https://delta.readthedocs.io/en/latest/usage/analysis.html>

**Reviewer #2:**

The authors introduce the second version of their python package: “Deep Learning for Time-lapse Analysis (DeLTA)”, which can be used to analyse microscopic images of bacteria, including segmentation and tracking over time in 2D. They use one U-Net to segment microscopy images and one U-Net to track single cells from frame to frame. To showcase the pipeline the authors analyze mixed populations of antibiotic resistant and susceptible cells by tracking pole age and growth rate across generations. This package can prove very useful for many researchers.

Major concerns:

Manuscript:
line 147: It would be interesting to see how the segmentation performs on an independent held out testset using common segmentation metrics, such as the dice score to evaluate how well the segmentation model performs. It is unclear to me if the segmentation model has systematic errors, such as over- or under-segmentations. As the authors claim that they quantify cell growth and cell length, the segmentation accuracy should be high. In Figure 3A it looks to me as if the model would under-segment the cells as the segmented cells look thinner than in the phase contrast image.
A scatter plot (or violin) of the error rate (line147) against the segmentation overlap (line 148/149) would be interesting to gain insight into the relationship between the two parameters.

We have addressed this by using common segmentation metrics to evaluate our test set. We now plot the intersection-over-union (IOU), Dice score, and error rate for each frame. In addition, we plot false positives, false negatives, and true positives + true negatives for a pixelwise analysis for errors over all frames in the movie.

We appreciate the reviewer’s concern about DeLTA under-segmenting the cells. We do find that DeLTA is more likely to under-segment than over-segment, as the reviewer predicted (S2B Fig). The segmentation model was trained with a high weight in order to identify the borders between the cells correctly (S7 Fig) as segmenting the borders becomes very difficult in crowded images. We are more interested in getting the length of the cells correct as opposed to getting the width, as this is the feature used to calculate growth rate. Although the ground truths were curated by an expert, the cell-border interface will never be perfectly segmented. As a result, we placed less weight there to allow the model to classify the cell-border interface. As shown in S2A Fig, the evaluation metrics are relatively constant throughout the movie, suggesting that results will be systematic across the movie. Since the under-segmentation is constant, morphological operations could be used to increase the area of the cells if this was a concern for the particular application.

🡪 See S2 Fig

Python package:

The authors should meet commonly used software standards for python packages to ensure long term usability of their tool:

Create a python package including version control using continuous numbering for every release

We have updated our repository and coding practices for better version control. We created a dev branch where we will make changes / bug fixes. When we merge the dev branch into the main branch, we will update the version number. Once the reviewers are satisfied with the manuscript revisions, we will create DeLTA v2.0.0 and perform proper version numbering from there with all future updates. We appreciate this comment and will implement this suggestion moving forward.

Provide a small example for users in the git repository so it is easy to initially run the package and gain understanding of how it works

Reviewer #1 made a similar comment, and we agree. As part of our test suite we incorporated evaluation datasets. However, these datasets are fairly small as git repositories should not be used to keep track of large amounts of data. Therefore, we also implemented a new DeLTA submodule, assets.py, that allows the user to selectively and automatically download our latest trained models and datasets. Now, after installation, the user can simply run delta.assets.download_assets() and either use our pipeline directly or check the example scripts to understand how our software works.

🡪 See <https://delta.readthedocs.io/en/latest/usage/assets_desc.html>

Implement unit tests (e.g. using pytest) with their pipeline to ensure quality control of their code and of future versions.

We have now adopted a workflow of systematic testing before merging branches, and have been developing unit and integration tests using pytest to test both the whole pipeline as well as separate modules and methods. The tests are available in the tests/ subfolder of our repository. We appreciate this suggestion and can see how this will save us a lot of time in the future.

🡪 See <https://gitlab.com/dunloplab/delta>

It would be helpful for many users to provide a jupyter-notebook, or google-colab-notebook, which users are guided through. (e.g. similar to:

<https://github.com/stardist/stardist/blob/master/examples/2D/3_prediction.ipynb>)

We agree, and note that Reviewer #1 made a similar request. We now provide a Colab notebook that allows the user to not only run the main pipeline on our evaluation data or their own time-lapse movies, but also to retrain the models on their own data. We mention the notebook in the text and the documentation.

🡪 See Colab notebook:

<https://colab.research.google.com/drive/1UL9oXmcJFRBAm0BMQy_DMKg4VHYGgtxZ>

Having a pip-installable version using pypi of the package would be great for many users and ease applications.

While we have usually had better luck installing dependencies with conda, we now provide DeLTA as a pip package (<https://pypi.org/project/delta2/>) and we also added a requirements file to our Gitlab. We also now provide a conda package on the conda-forge channel (<https://anaconda.org/conda-forge/delta2>).

🡪 See <https://delta.readthedocs.io/en/latest/usage/installation.html>

In order to help with installation issues, we also created a list of successful installations in the documentation.

🡪 See <https://delta.readthedocs.io/en/latest/usage/installs_lists.html>

Minor concerns:

line 62: “successive convolutional layers are progressively down-sampled”, not the layers are down-sampled, but the information is down-sampled using pooling layers.

We have changed the language to reflect that indeed it is information that is down-sampled, not the layers.

🡪 See Introduction

line 64: One major feature, which causes the UNet to perform well are the skip connections, which preserve fine details (high frequency information). I think the authors should mention this here.

We agree that skip connections are an important part of the U-Net architecture. We have added a description of the skip connections.

🡪 See Introduction

line 313: could computational cost be reduced by ignoring cells that have just divided, assuming a certain minimum time-distance between division events? Or by ignoring cells of a size smaller than a threshold (that is motivated biologically)?

After segmentation, we currently have an optional post-processing step that removes segmentations smaller than a certain threshold set by the config file.

Due to how DeLTA performs lineage evaluation, even if we “ignored” divisions for certain cells, we would still have to input the same number of tracking inputs into the model regardless, because both the operations of tracking and lineage attributions are performed by the same U-Net. It would most likely not change throughput, but it might improve tracking performance slightly. However, in order to add these rules, we would have to hard-code ad hoc post-processing conditions regarding the lineage reconstruction which would make the pipeline less adaptable. In general, we try to avoid using pre- and post-processing operations that involve additional parameters as much as possible.

🡪 See Introduction and Discussion

line 379: is the gaussian noise used for data augmentation only positive? When simulating microscopy noise, only additive gaussian noise should be considered. For further improvement lightning augmentations (contrast, illumination) could be added in a later release.

The Gaussian noise data augmentation is indeed additive. The distribution’s mean is zero and its standard deviation is set by the user. After noise addition, the pixel values are clipped back to the original dynamic range of the camera. At least on our setup, the noise distribution is very close to a zero-mean gaussian. We also incorporate a few lighting data augmentations that were inherited from DeLTA 1.0, such as image histogram deformations. The text has been updated to clarify these points.

🡪 See Methods section “Loss functions and training”

line 397: “For the segmentation training dataset, the initial segmentations were generated”, does this mean pixel-wise annotation by an expert?

The initial training set was generated by an expert utilizing Ilastik. We have clarified this in the text.

🡪 See Methods section “Training set generation and testing”

Figure 1A looks redundant with Figure 1B, C, D to me. Could be shortened?

We have simplified Fig. 1A to reduce redundancy.

🡪 See Fig. 1

All figures: Label sizes differ between subfigures, should be unified

Fixed.

Fig 1E, could all daughter cells of the one cell highlighted be plotted as well to emphasize the ability to track cells across division events?

We considered this, but since the movie starts with ~3 cells, tracking daughters with different colors ultimately includes a lot of the data and obscures the information for one cell so we ultimately decided to leave the image as is.

Other remarks:

Figure 1A: gray errors “Old Pole”, “New Pole” difficult to see.

With the new version of Fig. 1A we have corrected this.

Figure 2: Could the authors indicate which cutout of figure 2A is shown in Figure 2B, this would make the figure more comprehensive

We have made this change.

Figure 1A: Scale bar is hardly visible

Fixed.

line 78/79: “Click or tap here to enter text..“, should be removed

Fixed.

**Reviewer #3:**

The authors present an extension of their previous work on tracking and quantifying bacterial behavior via Deep Learning. The system relies on U-Nets for cell segmentation and for tracking and can be deployed for systematic large scale analyses of spatial and temporal dynamics of bacterial development in a 2D environment. The authors show two applications of their system for bacteria growing on 2D substrates. In particular they present an analysis demonstrating the impact of pole age on bacterial growth dynamics.

This is a good paper in principle. My major criticism would be the lack of details in describing the methods. While I see that it makes sense to reference earlier work here it might still be better to make the paper more self-contained by explaining e.g. the U-Net training (and loss functions) in more detail.

We thank the reviewer for these comments on how to make our work more accessible. We describe below how we have addressed these critiques by adding more details in the manuscript. We also added a supplementary figure on tracking as well as the loss function to help clarify some of the more complex methodological points.

For a broader audience it would also be important to discuss if this approach can be generalized to other applications of cell tracking or where the respective limitations are.

We have addressed this point in two ways. First, we have added text to clarify that this approach should be generalizable to other applications, potentially including single-cell tracking of other organisms. This is described in the Discussion. Second, we have added a supplementary movie (S2 Movie) showing DeLTA performance (without any retraining) on data from *B. subtilis* cells growing in a microfluidic device which we obtained from another lab.

🡪 See Discussion

🡪 See S2 Movie

Detailed comments see below:

[page 8]: (D) Cell tracking between frames. Representative examples of cell tracking with and without division are shown with a phase contrast image of the ‘previous frame’ on the left, a phase contrast image of the ‘current frame’ in the middle, and a grayscale image of the ‘prediction’ on the right. The ‘current frame’ shows the tracking prediction overlayed.

Please briefly describe the U-Net setup for the tracking in the methods section at least. What exactly are the inputs and outputs here?

We have added a new supplementary figure and text to address this question in more detail.

🡪 See S1 Fig

🡪 See Methods

[page 12]: (C) Growth rate within each generation.

What do the connecting lines mean in the right panel (showing great granddaughters). I am not sure I am interpreting this the right way.

The connecting lines show statistical significance. The caption for Fig. 3 has been updated to clarify this.

🡪 See Fig. 3C caption

[page 12]: (n = 10,726 cells; one-way ANOVA test performed; p-value ** ≤…

How did you locate the group differences? An ANOVA would only tell you that there is a difference somewhere, or maybe I am missing something here.

Thank you for pointing this out. You are correct, an ANOVA only tells you that there is a difference somewhere; a Tukey test is a post hoc test that tells you which groups are significantly different. In the caption for Fig 3, we have updated this to use the Tukey test.

🡪 See Fig. 3

[page 13]: Utilizing the U-Net convolutional neural network architecture, the model can rapidly segment and track cells frame-to-frame with a low error rate.

How is tracking done here?

We now reference the new S1 Fig for clarity.

🡪 See Fig. S1

[page 13]: coli, suggesting that analysis of cells with similar morphologies such as Bacillus, Pseudomonas, and Salmonella species will be straightforward.

Have you tested this claim by any chance?

Yes, in the revised manuscript we now show a complex movie with *B. subtilis* cells (S2 Movie). We also note for the reviewer’s reference that DeLTA has worked on *Salmonella* species and other organisms, but these images sets are currently unpublished so we do not reference them in the manuscript.

🡪 See S2 Movie

[page 13]: In addition, if the position of the cells within the field of view shifts dramatically from frame to frame, the current software does not perform well.

Do you have an estimate at least?

We have added a supplementary figure (S3 Fig) that details the tracking model’s accuracy with respect to shifts in the field of view.

🡪 See S3 Fig

[page 14]: recurrent neural networks could be combined with our models to improve segmentation by incorporating temporal context.

Have you tested his idea? How well does your method generalize to related tracking experiments?

We have not tested this idea. We updated the text in the manuscript to make it clear that this is a potential future direction.

🡪 See Discussion

[page 14]: Future efforts to optimize the tracking algorithm could help to address this by avoiding methods that scale linearly with the number of cells.

Any ideas here that could be worth mentioning?

We are currently working on this future direction, and while we have obtained some promising preliminary results, this new approach will still require development, testing, and benchmarking.

[page 14]: It works with many common microscopy file formats and extracts single-cell features such as cell poles, length, lineage, and fluorescence levels automatically and saves data into Python and Matlab compatible formats.

How easy is it for a user (say someone who can work with data and image analysis in Python) to adapt your system to other use-cases?

We have tried to make it easy for people to use DeLTA, though some programming skills are required. Users may clone the repository from Gitlab or install DeLTA through conda-forge or with pip. With this revision, we have also added extensive documentation that is available online.

🡪 See <https://delta.readthedocs.io/en/latest/>

🡪 See <https://delta.readthedocs.io/en/latest/usage/installation.html>

We also provide a suite of example scripts that are meant not only to demonstrate how to use our pipeline, but also to illustrate and explain how some key components of our software work.

🡪 See <https://delta.readthedocs.io/en/latest/usage/scripts.html>

We have also set up a Google Colab notebook for users to test DeLTA without needing to install DeLTA on their local machine.

🡪 See Colab notebook:

<https://colab.research.google.com/drive/1UL9oXmcJFRBAm0BMQy_DMKg4VHYGgtxZ>

Additionally, we have avoided implementing ad hoc pre- and post-processing operations as much as possible. The idea behind this is to have most of the work done by the two trainable U-Net models for segmentation and tracking. For that reason, we anticipate that upon re-training the models, it should be relatively easy to use DeLTA for different use-cases.

🡪 See Introduction and Discussion

Reference documentation and example files

[page 14]: As many microbiology researchers work with these types of data, we envision that this software can be used to increase the throughput of microscopy image analysis.

Given constraints on e.g. shape and frame rate it might be limited to a specific field I think. Could you comment on this or say to which scenarios you think the approach would generalize well?

In terms of segmentation, we think the current trained models will generalize well to rod-shaped bacteria, and we now demonstrate this with inclusion of the new S2 Movie. However, the trained models may not perform as well on more circular or irregularly shaped bacteria. A new model may need to be trained on these types of cells.

However, the core of DeLTA is the segmentation and tracking models which do not incorporate any hard-coded rules on shape or size. We have tried to make DeLTA as generalizable as possible and limit the rules in the lineage construction part as well. In fact, DeLTA 1.0 was tested on a small yeast dataset and performed relatively well given the small training set size. It was also adapted by another team to perform segmentation on yeast movies. We now include these ideas and references in the Discussion.

🡪 See Introduction and Discussion

In terms of tracking, images do need to be taken frequently enough as the model not only struggles with large cell movements or large shifts in the field of the view, but if time intervals are too large tracking and lineage reconstruction simply become impossible even manually. For *E. coli* cells growing in our conditions we suggest taking images every 5 minutes.

🡪 See Discussion

[page 14]: Network architecture for the segmentation and tracking model is as described in the original DeLTA publication…

I think it is worth doing a short recap of the networks here.

We have added more details about the neural networks used for the segmentation and tracking model.

🡪 See Methods section “Implementation and network architecture”

[page 14]: In DeLTA 2.0, the tracking model uses a sigmoid function as the final activation layer and a pixelwise-weighted binary cross-entropy loss function to produce a single grayscale output image with 1’s representing tracked cells (mother and potential daughter) and 0’s representing the background and the cells that did not track to the input cell.

What is the rationale or idea here?

We now include an additional supplementary figure showing the steps in the pixelwise-weighted binary cross-entropy loss function calculation (S6 Fig). Binary cross-entropy functions are standard in machine learning applications and have been adapted for use in U-Net architectures.

🡪 See S6 Fig

[page 15]: The segmentation model used to quantify the error rate was trained for 600 epochs with 300 steps per epoch and a batch size of 1.

Did you check convergence of the training?

Yes, all models converged during training. The training scripts for segmentation and tracking include logic where the models stop training if there are no updates in the last 50 epochs. In supplementary S9 Fig, we now show the loss for a segmentation model as a representative example.

🡪 See S9 Fig
