# Supplementary material for: DeLTA 2.0: A deep learning pipeline for quantifying single-cell spatial and temporal dynamics
Source: PLoS Comput Biol. 2022 Jan 18;18(1):e1009797. doi: 10.1371/journal.pcbi.1009797 (PMC8797229; doi:10.1371/journal.pcbi.1009797)
Supplement: Peer review history

# Peer review: Decision Letter 0

**DOI:** 10.1371/journal.pcbi.1009797.r001

**Authors:** Kiran Raosaheb Patil, Luis Pedro Coelho

29 Sep 2021

Dear Dr Dunlop,

Thank you very much for submitting your manuscript "DeLTA 2.0: A deep learning pipeline for quantifying single-cell spatial and temporal dynamics" for consideration at PLOS Computational Biology.

As with all papers reviewed by the journal, your manuscript was reviewed by members of the editorial board and by several independent reviewers. In light of the reviews (below this email), we would like to invite the resubmission of a significantly-revised version that takes into account the reviewers' comments.

All three reviewers are generally positive about the work. However, not only do they point to several instances where there is a lack of clarity and detail, but there is also general agreement between the reviewers that additional benchmarking or contextualization of the results (what are the strengths and limitations of the approaches taken) would make the manuscript's claims more grounded. I'll also note that two reviewers independently mention issues regarding installation and suggest a google collab (or equivalent) addition to the extant documentation.

We cannot make any decision about publication until we have seen the revised manuscript and your response to the reviewers' comments. Your revised manuscript is also likely to be sent to reviewers for further evaluation.

When you are ready to resubmit, please upload the following:

[1] A letter containing a detailed list of your responses to the review comments and a description of the changes you have made in the manuscript. Please note while forming your response, if your article is accepted, you may have the opportunity to make the peer review history publicly available. The record will include editor decision letters (with reviews) and your responses to reviewer comments. If eligible, we will contact you to opt in or out.

[2] Two versions of the revised manuscript: one with either highlights or tracked changes denoting where the text has been changed; the other a clean version (uploaded as the manuscript file).

Important additional instructions are given below your reviewer comments.

Please prepare and submit your revised manuscript within 60 days. If you anticipate any delay, please let us know the expected resubmission date by replying to this email. Please note that revised manuscripts received after the 60-day due date may require evaluation and peer review similar to newly submitted manuscripts.

Thank you again for your submission. We hope that our editorial process has been constructive so far, and we welcome your feedback at any time. Please don't hesitate to contact us if you have any questions or comments.

Sincerely,

Luis Pedro Coelho

Associate Editor

PLOS Computational Biology

Kiran Patil

Deputy Editor

PLOS Computational Biology

***********************

All three reviewers are generally positive about the work. However, not only do they point to several instances where there is a lack of clarity and detail, but there is also general agreement between the reviewers that additional benchmarking or contextualization of the results (what are the strengths and limitations of the approaches taken) would make the manuscript's claims more grounded. I'll also note that two reviewers independently mention issues regarding installation and suggest a google collab (or equivalent) addition to the extant documentation.

Reviewer's Responses to Questions

**Comments to the Authors:**

**Please note here if the review is uploaded as an attachment.**

Reviewer #1: O’Connor et al describe DeLTA 2.0, a cell-segmentation, tracking, and fluorescence data analysis pipeline which improves upon the original version of DeLTA in several significant ways. In the introduction, the authors note that increasing data throughput requires reducing time spent on manually correcting segmentation and tracking. This undersells the potential impact of this work, which does not require particularly high throughput data to save many hours. There are also experiments beyond timelapse fluorescence microscopy of bacteria that the DeLTA 2.0 data pipeline can be modified to cover. And some examples the authors cite include a constitutive fluorescence signal to assist in segmentation, which is a complication that can hopefully be avoided with DeLTA.

In addition to creating an end-to-end pipeline from raw data to fluorescence for cell lineages, DeLTA 2.0 makes improvements to segmentation training that increase training speed and accuracy. The authors show that the network can be reduced in size without much impact on performance, which would increase the number of potential users with mobile GPUs, for example, or allow for larger images to be processed without tiling.

As with DeLTA 1.0, in my hands DeLTA 2.0 must be trained on our particular data sets to achieve usable segmentation; despite having a similar resolution, a reduction in contrast in our images requires retraining the network. This is in contrast with MiSiC (DOI: 10.7554/eLife.65151) that applies a shape index map to all training and prediction inputs to achieve a degree of generalization. In future work, it would be interesting to see whether a DeLTA 2.0 model trained on shape index maps would be sufficient to generate initial training data.

For many labs the barrier to using DeLTA will be in generating training data and adjusting training parameters. In the readme file the authors write, “We are currently working on the implementation graphical interfaces to generate training sets and to analyze and correct delta outputs,” which will be important, especially for those without Matlab.

Specific comments:

* Line 141: “DeLTA 2.0 can process datasets of any dimension quickly” is presumably not true at some limit. A brief discussion of system and GPU memory requirements and likely performance on a common consumer-grade processor and GPU would be useful. On a PC with 16 GB of system memory I ran into some issues for a large example file.

* Line 193: The addition of noise is also a feature of MiSiC - 10.7554/eLife.65151 - which could be discussed further. With DeLTA 1.0, I found that the inverse operation also worked – applying a low-pass filter to training and test data improved performance, especially for segmenting objects thinner than E. coli cells. My impression was that this occurred because some data augmentation operations effectively do this by interpolating pixels, so most training data was unlike test data.

* Figure 2 and Figure S1: I do not see any need for Fig. S1; the difference in growth rates is very clear in Fig. 2D as well as Fig. A. Also, the unlabeled cells in the “Susceptible” panel of Fig. 2B appear to be “Resistant” and perhaps it would be more clear to show only this microcolony, highlighting the green “Susceptible” cell and purple “Resistant” cells in all frames.

General minor comments:

* The authors could briefly compare to DeepCell - 10.1371/journal.pcbi.1005177 - which also describes E. coli segmentation and tracking.

* Installation on Windows is not straightforward, but is described well enough in the readme to manage. Listing specific versions in the .yml file would have been helpful, and perhaps installation can be simplified for users not needing bioformats.

* The file including models, example data, and training data is very large. Separate files would be useful--especially a small file of example data.

* More people will try DeLTA if there is a Google Colab notebook or similar that eliminates needing to sort out system configuration; it would be valuable to have one example notebook for training and one example notebook for segmentation, tracking, and displaying some figures of timelapse data similar to those in the paper.

Reviewer #2: Summary:

The authors introduce the second version of their python package: “Deep Learning for Time-lapse Analysis (DeLTA)”, which can be used to analyse microscopic images of bacteria, including segmentation and tracking over time in 2D. They use one U-Net to segment microscopy images and one U-Net to track single cells from frame to frame. To showcase the pipeline the authors analyze mixed populations of antibiotic resistant and susceptible cells by tracking pole age and growth rate across generations. This package can prove very useful for many researchers.

Major concerns:

Manuscript:

line 147: It would be interesting to see how the segmentation performs on an independent held out testset using common segmentation metrics, such as the dice score to evaluate how well the segmentation model performs. It is unclear to me if the segmentation model has systematic errors, such as over- or under-segmentations. As the authors claim that they quantify cell growth and cell length, the segmentation accuracy should be high. In Figure 3A it looks to me as if the model would under-segment the cells as the segmented cells look thinner than in the phase contrast image.

A scatter plot (or violin) of the error rate (line147) against the segmentation overlap (line 148/149) would be interesting to gain insight into the relationship between the two parameters.

Python package:

The authors should meet commonly used software standards for python packages to ensure long term usability of their tool:

Create a python package including version control using continuous numbering for every release

Provide a small example for users in the git repository so it is easy to initially run the package and gain understanding of how it works

Implement unit tests (e.g. using pytest) with their pipeline to ensure quality control of their code and of future versions.

It would be helpful for many users to provide a jupyter-notebook, or google-colab-notebook, which users are guided through. (e.g. similar to: https://github.com/stardist/stardist/blob/master/examples/2D/3_prediction.ipynb)

Having a pip-installable version using pypi of the package would be great for many users and ease applications.

Minor concerns:

line 62: “successive convolutional layers are progressively down-sampled”, not the layers are down-sampled, but the information is down-sampled using pooling layers.

line 64: One major feature, which causes the UNet to perform well are the skip connections, which preserve fine details (high frequency information). I think the authors should mention this here.

line 313: could computational cost be reduced by ignoring cells that have just divided, assuming a certain minimum time-distance between division events? Or by ignoring cells of a size smaller than a threshold (that is motivated biologically)?

line 379: is the gaussian noise used for data augmentation only positive? When simulating microscopy noise, only additive gaussian noise should be considered. For further improvement lightning augmentations (contrast, illumination) could be added in a later release.

line 397: “For the segmentation training dataset, the initial segmentations were generated”, does this mean pixel-wise annotation by an expert?

Figure 1A looks redundant with Figure 1B, C, D to me. Could be shortened?

All figures: Label sizes differ between subfigures, should be unified

Fig 1E, could all daughter cells of the one cell highlighted be plotted as well to emphasize the ability to track cells across division events?

Other remarks:

Figure 1A: gray errors “Old Pole”, “New Pole” difficult to see.

Figure 2: Could the authors indicate which cutout of figure 2A is shown in Figure 2B, this would make the figure more comprehensive

Figure 1A: Scale bar is hardly visible

line 78/79: “Click or tap here to enter text..“, should be removed

Reviewer #3: The authors present an extension of their previous work on tracking and quantifying bacterial behavior via Deep Learning. The system relies on U-Nets for cell segmentation and for tracking and can be deployed for systematic large scale analyses of spatial and temporal dynamics of bacterial development in a 2D environment. The authors show two applications of their system for bacteria growing on 2D substrates. In particular they present an analysis demonstrating the impact of pole age on bacterial growth dynamics.

This is a good paper in principle. My major criticism would be the lack of details in describing the methods. While I see that it makes sense to reference earlier work here it might still be better to make the paper more self-contained by explaining e.g. the U-Net training (and loss functions) in more detail.

For a broader audience it would also be important to discuss if this approach can be generalized to other applications of cell tracking or where the respective limitations are.

Detailed comments see below:

[page 8]: (D) Cell tracking between frames. Representative examples of cell tracking with and without division are shown with a phase contrast image of the ‘previous frame’ on the left, a phase contrast image of the ‘current frame’ in the middle, and a grayscale image of the ‘prediction’ on the right. The ‘current frame’ shows the tracking prediction overlayed.

Please briefly describe the U-Net setup for the tracking in the methods section at least. What exactly are the inputs and outputs here?

[page 12]: (C) Growth rate within each generation.

What do the connecting lines mean in the right panel (showing great granddaughters). I am not sure I am interpreting this the right way.

[page 12]: (n = 10,726 cells; one-way ANOVA test performed; p-value ** ≤…

How did you locate the group differences? An ANOVA would only tell you that there is a difference somewhere, or maybe I am missing something here.

[page 13]: Utilizing the U-Net convolutional neural network architecture, the model can rapidly segment and track cells frame-to-frame with a low error rate.

How is tracking done here?

[page 13]: coli, suggesting that analysis of cells with similar morphologies such as Bacillus, Pseudomonas, and Salmonella species will be straightforward.

Have you tested this claim by any chance?

[page 13]: In addition, if the position of the cells within the field of view shifts dramatically from frame to frame, the current software does not perform well.

Do you have an estimate at least?

[page 14]: recurrent neural networks could be combined with our models to improve segmentation by incorporating temporal context.

Have you tested his idea? How well does your method generalize to related tracking experiments?

[page 14]: Future efforts to optimize the tracking algorithm could help to address this by avoiding methods that scale linearly with the number of cells.

Any ideas here that could be worth mentioning?

[page 14]: It works with many common microscopy file formats and extracts single-cell features such as cell poles, length, lineage, and fluorescence levels automatically and saves data into Python and Matlab compatible formats.

How easy is it for a user (say someone who can work with data and image analysis in Python) to adapt your system to other use-cases?

[page 14]: As many microbiology researchers work with these types of data, we envision that this software can be used to increase the throughput of microscopy image analysis.

Given constraints on e.g. shape and frame rate it might be limited to a specific field I think. Could you comment on this or say to which scenarios you think the approach would generalize well?

[page 14]: Network architecture for the segmentation and tracking model is as described in the original DeLTA publication…

I think it is worth doing a short recap of the networks here.

[page 14]: In DeLTA 2.0, the tracking model uses a sigmoid function as the final activation layer and a pixelwise-weighted binary cross-entropy loss function to produce a single grayscale output image with 1’s representing tracked cells (mother and potential daughter) and 0’s representing the background and the cells that did not track to the input cell.

What is the rationale or idea here?

[page 15]: The segmentation model used to quantify the error rate was trained for 600 epochs with 300 steps per epoch and a batch size of 1.

Did you check convergence of the training?

**********

**Have the authors made all data and (if applicable) computational code underlying the findings in their manuscript fully available?**

The PLOS Data policy requires authors to make all data and code underlying the findings described in their manuscript fully available without restriction, with rare exception (please refer to the Data Availability Statement in the manuscript PDF file). The data and code should be provided as part of the manuscript or its supporting information, or deposited to a public repository. For example, in addition to summary statistics, the data points behind means, medians and variance measures should be available. If there are restrictions on publicly sharing data or code —e.g. participant privacy or use of data from a third party—those must be specified.

Reviewer #1: Yes

Reviewer #2: Yes

Reviewer #3: Yes

**********

PLOS authors have the option to publish the peer review history of their article (what does this mean?). If published, this will include your full peer review and any attached files.

If you choose “no”, your identity will remain anonymous but your review may still be made public.

**Do you want your identity to be public for this peer review?** For information about this choice, including consent withdrawal, please see our Privacy Policy.

Reviewer #1: **Yes: **Zach Hensel

Reviewer #2: **Yes: **Dominik Waibel

Reviewer #3: No

Figure Files:

While revising your submission, please upload your figure files to the Preflight Analysis and Conversion Engine (PACE) digital diagnostic tool, https://pacev2.apexcovantage.com. PACE helps ensure that figures meet PLOS requirements. To use PACE, you must first register as a user. Then, login and navigate to the UPLOAD tab, where you will find detailed instructions on how to use the tool. If you encounter any issues or have any questions when using PACE, please email us at figures@plos.org.

Data Requirements:

Please note that, as a condition of publication, PLOS' data policy requires that you make available all data used to draw the conclusions outlined in your manuscript. Data must be deposited in an appropriate repository, included within the body of the manuscript, or uploaded as supporting information. This includes all numerical values that were used to generate graphs, histograms etc.. For an example in PLOS Biology see here: http://www.plosbiology.org/article/info%3Adoi%2F10.1371%2Fjournal.pbio.1001908#s5.

Reproducibility:

To enhance the reproducibility of your results, we recommend that you deposit your laboratory protocols in protocols.io, where a protocol can be assigned its own identifier (DOI) such that it can be cited independently in the future. Additionally, PLOS ONE offers an option to publish peer-reviewed clinical study protocols. Read more information on sharing protocols at https://plos.org/protocols?utm_medium=editorial-email&utm_source=authorletters&utm_campaign=protocols

# Author comment: Author response to Decision Letter 0

**DOI:** 10.1371/journal.pcbi.1009797.r002

30 Nov 2021

Attachment Submitted filename: Response_to_Reviewers.docx Click here for additional data file.

# Peer review: Decision Letter 1

**DOI:** 10.1371/journal.pcbi.1009797.r003

**Authors:** Kiran Raosaheb Patil, Luis Pedro Coelho

25 Dec 2021

Dear Dr Dunlop,

We are pleased to inform you that your manuscript 'DeLTA 2.0: A deep learning pipeline for quantifying single-cell spatial and temporal dynamics' has been provisionally accepted for publication in PLOS Computational Biology.

Before your manuscript can be formally accepted you will need to complete some formatting changes, which you will receive in a follow up email. A member of our team will be in touch with a set of requests.

Please note that your manuscript will not be scheduled for publication until you have made the required changes, so a swift response is appreciated.

IMPORTANT: The editorial review process is now complete. PLOS will only permit corrections to spelling, formatting or significant scientific errors from this point onwards. Requests for major changes, or any which affect the scientific understanding of your work, will cause delays to the publication date of your manuscript.

Should you, your institution's press office or the journal office choose to press release your paper, you will automatically be opted out of early publication. We ask that you notify us now if you or your institution is planning to press release the article. All press must be co-ordinated with PLOS.

Thank you again for supporting Open Access publishing; we are looking forward to publishing your work in PLOS Computational Biology. 

Best regards,

Luis Pedro Coelho

Associate Editor

PLOS Computational Biology

Kiran Patil

Deputy Editor

PLOS Computational Biology

***********************************************************

The reviewers recommend accepting the paper and agree that the manuscript and tool are valuable for the community. They have a few comments, which the authors may want to take into consideration for their future work (and perhaps there is a small coding issue that could be addressed over the short term), but none that would preclude publication.

Reviewer's Responses to Questions

**Comments to the Authors:**

**Please note here if the review is uploaded as an attachment.**

Reviewer #1: I think that the authors sufficiently addressed all issues that were raised in review. DeLTA 2.0 is certainly something we will use in our lab, and changes described in this revision that make it easier to adopt mean that more labs will use it. I tested the Colab pipeline implementation and found that it worked well after commenting out two lines that were giving an error (91: elif self.reader.resfolder is not None:, and 92: self.resfolder = self.reader.resfolder).

For sensitivity to global drift (lines 218-224), we have found that preprocessing after segmentation to estimate and correct drift from the center of mass of segmented objects works well. However, this approach might not be easily generalizable to data sets with cells entering/exiting the image and only works for global shifts in object positions.

Reviewer #2: I thank the authors for their improvements on the manuscript and repository and think their work will be very beneficial for the community. Without further comments I recommend this manuscript for publication. No further review is uploaded as an attachment.

Reviewer #3: Thanks for revising the manuscript. I particularly appreciate the improved documentation and examples. I am convinced this will be very helpful for the community.

I think there was a misunderstanding regarding my question about the tracking model and binary classification loss. My question was: What is the motivating idea for posing the tracking question as a binary segmentation problem at the pixel level? But I think this is not a critical point to discuss. Sorry for not phrasing the question precisely enough.

**********

**Have the authors made all data and (if applicable) computational code underlying the findings in their manuscript fully available?**

The PLOS Data policy requires authors to make all data and code underlying the findings described in their manuscript fully available without restriction, with rare exception (please refer to the Data Availability Statement in the manuscript PDF file). The data and code should be provided as part of the manuscript or its supporting information, or deposited to a public repository. For example, in addition to summary statistics, the data points behind means, medians and variance measures should be available. If there are restrictions on publicly sharing data or code —e.g. participant privacy or use of data from a third party—those must be specified.

Reviewer #1: Yes

Reviewer #2: Yes

Reviewer #3: Yes

**********

PLOS authors have the option to publish the peer review history of their article (what does this mean?). If published, this will include your full peer review and any attached files.

If you choose “no”, your identity will remain anonymous but your review may still be made public.

**Do you want your identity to be public for this peer review?** For information about this choice, including consent withdrawal, please see our Privacy Policy.

Reviewer #1: **Yes: **Zach Hensel

Reviewer #2: **Yes: **Dominik Waibel

Reviewer #3: **Yes: **Nico Scherf

# Editor report: Acceptance letter

**DOI:** 10.1371/journal.pcbi.1009797.r004

**Authors:** Kiran Raosaheb Patil, Luis Pedro Coelho

12 Jan 2022

PCOMPBIOL-D-21-01464R1 

DeLTA 2.0: A deep learning pipeline for quantifying single-cell spatial and temporal dynamics

Dear Dr Dunlop,

I am pleased to inform you that your manuscript has been formally accepted for publication in PLOS Computational Biology. Your manuscript is now with our production department and you will be notified of the publication date in due course.

The corresponding author will soon be receiving a typeset proof for review, to ensure errors have not been introduced during production. Please review the PDF proof of your manuscript carefully, as this is the last chance to correct any errors. Please note that major changes, or those which affect the scientific understanding of the work, will likely cause delays to the publication date of your manuscript. 

Soon after your final files are uploaded, unless you have opted out, the early version of your manuscript will be published online. The date of the early version will be your article's publication date. The final article will be published to the same URL, and all versions of the paper will be accessible to readers.

Thank you again for supporting PLOS Computational Biology and open-access publishing. We are looking forward to publishing your work! 

With kind regards,

Zsofia Freund

PLOS Computational Biology | Carlyle House, Carlyle Road, Cambridge CB4 3DN | United Kingdom ploscompbiol@plos.org | Phone +44 (0) 1223-442824 | ploscompbiol.org | @PLOSCompBiol
